# *Candida auris* as an emerging threat in intensive care units: A single-center retrospective study

**DOI:** 10.1016/j.ijregi.2026.100865

**Published:** 2026-02-21

**Authors:** Bülent Kaya, Serap Demir Tekol, Elif Bombacı, Recep Demirhan

**Affiliations:** 1 Department of Infectious Diseases and Clinical Microbiology, University of Health Sciences, Kartal Dr Lütfi Kırdar City Hospital, Istanbul, Türkiye; 2 Department Microbiology, University of Health Sciences, Kartal Dr Lütfi Kırdar City Hospital, Istanbul, Türkiye; 3 Department of Anesthesiology and Reanimation, University of Health Sciences, Kartal Dr Lütfi Kırdar City Hospital, Istanbul, Türkiye; 4 Department of Thoracic Surgery, University of Health Sciences, Kartal Dr Lütfi Kırdar City Hospital, Istanbul, Türkiye

**Keywords:** *Candida auris*, Antifungal drug resistance, Bloodstream infections, Nosocomial infections, Intensive care unit

## Abstract

**Objectives:**

*Candida auris* is an emerging multidrug-resistant fungal pathogen associated with health care–associated outbreaks, particularly, in intensive care units, and is increasingly recognized as a cause of invasive bloodstream infections worldwide.

**Methods:**

This retrospective single-center study analyzed *C. auris* isolates obtained from blood cultures of 31 patients admitted to the mixed intensive care units of a tertiary multidisciplinary training and research hospital between December 2021 and December 2024. Demographic characteristics, underlying comorbidities, antifungal susceptibility profiles, treatment strategies, and in-hospital outcomes were systematically evaluated using hospital information system records.

**Results:**

The median age was 61 years (range, 21-87), and 55% were male. Fluconazole resistance was detected in 60% (18 of 30) of isolates. Automated susceptibility testing indicated elevated amphotericin B minimum inhibitory concentration values in all isolates; however, the reliability of automated methods for amphotericin B testing in *C. auris* remains controversial. All isolates remained susceptible to caspofungin and micafungin, and voriconazole susceptibility was inferred based on fluconazole minimum inhibitory concentration values. Echinocandins were used as first-line therapy. In eight (25.8%) patients, *C. auris* was identified in blood cultures after patient death.

**Conclusions:**

After the identification of *C. auris*, this institution implemented enhanced infection control measures, including contact isolation, environmental decontamination, and reinforcement of antifungal stewardship. Despite these interventions, delayed detection and limited access to reference susceptibility testing remained major challenges. These findings highlight the need for strengthened active surveillance, rapid diagnostic capacity, and standardized susceptibility testing to reduce nosocomial transmission and improve clinical outcomes in high-risk intensive care settings.

## Highlights

• *Candida auris* causes high mortality in critically ill patients in the intensive care unit.
• Fluconazole resistance is frequent, and echinocandins remain active.
• Delayed detection impairs effective infection control.
• Prolonged intensive care unit stay and invasive care drive acquisition.
• Enhanced surveillance is essential to limit hospital spread.

## Introduction

*Candida auris* is an emerging hospital-acquired yeast species that was first described in Japan in 2009 and has since demonstrated rapid global dissemination [1]. Whole genome sequencing analyses of *C. auris* strains isolated from different geographic regions have shown that the pathogen did not originate from a single source but rather emerged independently in at least four distinct locations, forming six major clades. These clades differ by tens of thousands of single nucleotide polymorphisms (SNPs). The identified clades include the East Asian clade (clade I), the South Asian clade (clade II), the South American clade (clade III), and the South African clade (clade IV) [2,3]. In addition, the Iranian clade V and a new lineage of Southeast Asian/Malaysian origin are considered as clade VI. This clade was detected in a patient without a history of international travel and differs from the other clades by more than 200,000 SNPs [4].

One possible explanation for the near-simultaneous emergence of this yeast across multiple regions is the increased selective pressure exerted by widespread antifungal use in agriculture, as well as in human and veterinary medicine. In addition, climate change has been proposed as a contributing factor, facilitating the adaptation of *C. auris* to human body temperature and enhancing its capacity to cause human infection [5].

Clinical *C. auris* isolates have been recovered from a wide range of specimens, including sterile body fluids, respiratory tract samples, urine, bile, tissue specimens, wound samples, and central venous catheters [6]. The first reported case in Türkiye was identified by Kurt et al. [7] in March 2021, followed by additional cases reported from Istanbul and Izmir.

Due to its multidrug resistance, diagnostic and therapeutic challenges, and high transmissibility within health care settings, *C. auris* has been classified as an urgent threat by the US Centers for Disease Control and Prevention (CDC) and was listed among the fungal pathogens of critical priority by the World Health Organization in 2022 [8]. The pathogen is capable of colonizing the skin, mucosa, and various environmental surfaces in health care environments. Its ability to persist on dry surfaces outside the host substantially contributes to the development of hospital outbreaks [9].

Risk factors for *C. auris* colonization and infection include prolonged exposure to health care environments, invasive procedures, and underlying comorbidities [10]. Patients who are immunocompromised and those with diabetes mellitus, hypertension, chronic pulmonary or renal disease, recent surgical interventions, parenteral nutrition, urinary or central venous catheters, exposure to broad-spectrum antimicrobial and antifungal agents, mechanical ventilation, and prolonged intensive care unit (ICU) stays are at increased risk for fungal infections [11,12].

For antifungal susceptibility testing, the Clinical and Laboratory Standards Institute and the European Committee on Antimicrobial Susceptibility Testing recommend the microdilution reference method. Although susceptibility break points have been established for Candida species, *C. auris*–specific break points have not yet been clearly defined [13]. However, based on expert opinion and data from closely related Candida species, the CDC has proposed provisional break points for fluconazole, amphotericin B, and echinocandins [14]. The CDC does not recommend antifungal treatment for patients colonized with *C. auris* in the absence of clinical signs or symptoms of infection [8].

Therefore, this study aimed to describe the clinical characteristics, antifungal susceptibility patterns, and treatment approaches of *C. auris* bloodstream infections in a tertiary-care intensive care setting in Türkiye. By providing institution-based epidemiologic and resistance data, this study seeks to contribute to regional surveillance efforts, inform infection prevention strategies, and support antifungal stewardship programs in the context of this emerging global health care threat.

## Materials and methods

This retrospective, single-center observational study included *C. auris* isolates obtained from blood cultures of 31 patients admitted to the mixed ICUs of a multidisciplinary training and research hospital between December 2021 and December 2024. Demographic characteristics, antifungal resistance profiles of *C. auris* isolates recovered from blood cultures, ICU length of stay, time from ICU admission to detection of *C. auris* in blood cultures, underlying comorbidities, previous surgical interventions, exposure to antibiotic therapy, and in-hospital mortality were retrieved from the hospital information system and systematically recorded in a standardized electronic database.

Patients with repeated *C. auris* isolations from the same episode, pregnant women, and individuals younger than 18 years were excluded from the study.

The study was conducted in the microbiology laboratory of our city hospital, where the infection control committee is actively working. Blood culture bottles were incubated for up to 5 days using the BacT/Alert 3D system (bioMérieux, Marcy-l’Étoile, France). Bottles signaling microbial growth were subjected to Gram staining, and aliquots were simultaneously subcultured onto Sabouraud dextrose agar, MacConkey agar, chocolate agar, and 5% sheep blood agar (bioMérieux, Marcy-l’Étoile, France). Plates were incubated at 35-37°C and examined after 18-24 hours. Species-level identification was performed using matrix-assisted laser desorption/ionization time-of-flight mass spectrometry (MALDI-TOF MS) (VITEK MS, bioMérieux, Marcy-l’Étoile, France).

Antifungal susceptibility testing was conducted using the VITEK 2 Compact system (bioMérieux, Marcy-l’Étoile, France) with AST YS08 cards, in accordance with the manufacturer’s instructions. Minimum inhibitory concentration (MIC) values were interpreted using the provisional breakpoints proposed by the CDC: fluconazole ≥32 µg/ml, amphotericin B ≥2 µg/ml, caspofungin ≥2 µg/ml, and micafungin ≥4 µg/ml were considered resistant. Voriconazole susceptibility was inferred based on fluconazole MIC values [15].

Descriptive statistical analyses were performed using standard statistical software. Continuous variables were expressed as medians with ranges and categorical variables as frequencies and percentages.

The study protocol was reviewed and approved by the institutional ethics committee, and the requirement for informed consent was waived due to the retrospective nature of the study.

### Statistical analysis

The distribution of variables was summarized as counts (n) and percentages (%) for categorical variables and as means with SDs or medians with ranges (minimum to maximum) for continuous variables, as appropriate. The normality of continuous variables was assessed using the Shapiro–Wilk test. Comparisons between groups, when applicable, were performed using the Student’s *t*-test or the Mann-Whitney *U* test for continuous variables and the chi-square test or Fisher’s exact test for categorical variables. A two-tailed *P* <0.05 was considered statistically significant. All statistical analyses were conducted using SPSS software (version 26.0; IBM Corp., Armonk, NY, USA).

## Results

A total of 31 patients were included in the analysis. The median age was 61 years (range, 21-87), with 17 (55%) male and 14 (45%) female patients (Table 1). All patients had received broad-spectrum antibiotics before the isolation of *C. auris* from blood cultures.

**Table 1:** Demographic and clinical characteristics of patients with *Candida auris* bloodstream infection (N = 31).

Variable	Median (Range)	n (%)
Age (years)	61 (21-87)	-
Female sex	-	14 (45%)
Male sex	-	17 (55%)
In-hospital mortality	-	18 (58%)
ICU length of stay (days)	44 (7-146)	-
Total ICU patient-days	-	1360
Time to blood culture positivity (days)	32 (6-75)	-

ICU, intensive care unit.

Note: Data are presented as median (range) or percentage (%), as appropriate.

ICU days: Patient monitoring periods in intensive care unit.

Blood culture days: Time from ICU admission to blood culture collection.

Fluconazole resistance was detected in 60% (18 of 30) of isolates, whereas all isolates exhibited resistance to amphotericin B. In contrast, all isolates remained susceptible to caspofungin and micafungin, and voriconazole susceptibility was inferred based on fluconazole MIC values (Table 2).

**Table 2:** MIC distribution of *Candida auris* isolates by antifungal agent (µg/mL).

Antifungal agent	≤0.012	≤0.06	0.12	0.25	0.5	2	4	8	16	≥16	32
**Fluconazole** **(n = 30)**	–	–	–	–	–	–	–	–	12 (40.0)	–	18 (60.0)
**Caspofungin** **(n = 31)**	1 (3.2)	–	3 (9.7)	25 (80.6)	2 (6.5)	–	–	–	–	–	–
**Micafungin** **(n = 30)**	–	15 (50.0)	12 (40.0)	3 (10.0)	–	–	–	–	–	–	–
**Anidulafungin** **(n = 4)**	–	–	3 (75.0)	1 (25.0)	–	–	–	–	–	–	–
**Voriconazole** **(n = 31)**	17 (54.8)	–	7 (22.6)	7 (22.6)	–	–	–	–	–	–	–
**Amphotericin B** **(n = 31)**	–	–	–	–	–	8 (25.8)	5 (16.1)	15 (48.4)	–	3 (9.7)	–

MIC, minimum inhibitory concentration.

Values represent number of isolates at each MIC concentration.

Break points were interpreted according to US Centers for Disease Control and Prevention provisional criteria for *Candida auris*.

Susceptibility testing was performed using the VITEK 2 automated system.

Echinocandins were used as first-line therapy in the majority of patients, with caspofungin initiated in 22 (71.0%) cases using a loading dose of 70 mg intravenously, followed by 50 mg once daily. One (3.2%) patient received voriconazole. In eight (25.8%) patients, *C. auris* was identified in blood cultures postmortem, precluding the initiation of antifungal therapy.

**Figure 1:**
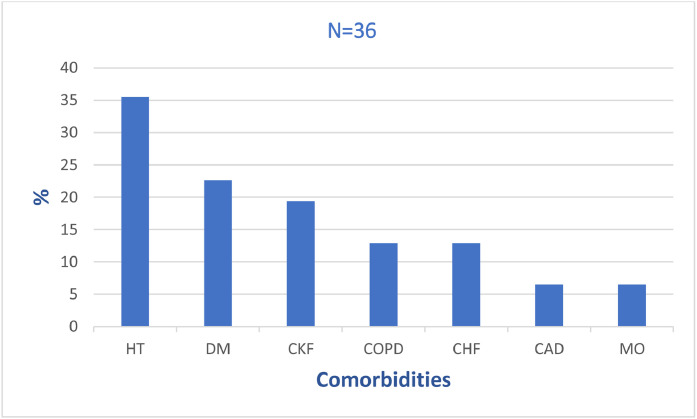
Comorbidities among patients with *Candida auris* bloodstream infection identified in a tertiary urban hospital over a 3-year period (N = 36).

CAD, coronary artery disease (n = 2); CHF, congestive heart failure (n = 4); CKF, chronic kidney failure (n = 6), COPD, chronic obstructive pulmonary disease (n = 4); DM, diabetes mellitus (n = 7); HT, hypertension (n = 11); MO, morbidly obese (n = 2).

Multiple comorbidities were observed, most commonly, hypertension (35.5%, n = 11), diabetes mellitus (22.6%, n = 7), and chronic kidney disease (19.4%, n = 6) (Figure 1).

**Figure 2:**
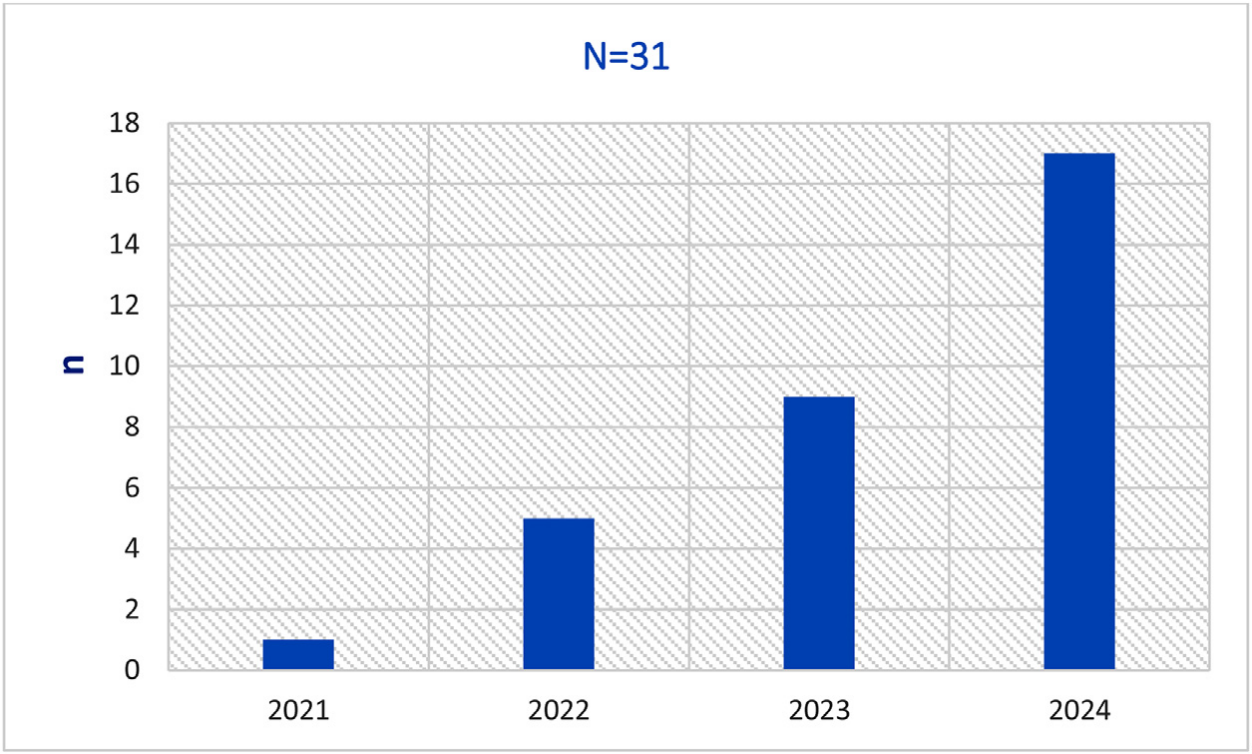
Annual distribution of *Candida auris* cases identified in the study hospital, 2021-2024 (N = 31).

The annual distribution of cases demonstrated a progressive increase in *C. auris* isolation over the study period, with the highest number of cases recorded in 2024 (Figure 2). The cumulative ICU length of stay was 1360 patient-days (range, 7-146 days per patient), and *C. auris* was detected at a median of 32 days after ICU admission (range, 6-75 days). The overall in-hospital mortality rate was 60% (18 of 30) (Table 1).

Major surgical procedures had been performed in 11 (35.5%) patients. These included total abdominal hysterectomy (n = 2), gastrectomy, gastrostomy, total hip arthroplasty, pancreatectomy, esophagectomy, cardiac stenting, corneal surgery, multiple fracture surgery, and lower extremity amputation. It was common for individuals to have received antibiotic treatment before *C. auris* was isolated, with meropenem (74.2%), tigecycline (58.0%), polymyxin E (41.9%), and teicoplanin (41.9%) being the most frequently administered agents.

## Discussion

*C. auris* has emerged as a formidable nosocomial pathogen and a major challenge for modern health care systems, particularly, within ICUs where critically ill and immunocompromised patients are concentrated. Its rapid global dissemination, persistence in the hospital environment, and propensity for multidrug resistance distinguish it from other *Candida* species and place it at the intersection of clinical mycology, infection prevention, and antimicrobial stewardship.

The temporal association between the first detection of *C. auris* in this institution and the COVID-19 pandemic mirrors observations from multiple regions, where disruptions in infection control practices, increased use of broad-spectrum antimicrobials, and unprecedented ICU overcrowding have been implicated in facilitating transmission. Alanazi et al. highlighted how strained health care infrastructures and reallocation of infection prevention resources during the pandemic may have inadvertently created ecological niches favoring the establishment and spread of *C. auris* [16]. Our findings support this hypothesis because the progressive increase in isolates over subsequent years suggests sustained endemicity rather than sporadic introduction.

From an epidemiologic standpoint, the annual distribution of cases in our cohort demonstrates a clear and progressive increase in *C. auris* isolation over the study period, culminating in the highest case burden in 2024 (Figure 2). This temporal trend is consistent with reports from regional surveillance networks indicating a transition from sporadic introductions to sustained health care–associated transmission within institutional settings [17,18]. The observed escalation suggests the establishment of local transmission chains rather than repeated importation events, highlighting the importance of continuous surveillance and longitudinal monitoring to detect early signals of endemicity.

Demographic patterns observed in our cohort are consistent with previous regional and international reports. Although male predominance and advanced age have been frequently described, the absence of a clear biological rationale for sex as an independent risk factor underscores the likelihood that these associations reflect underlying exposure patterns, comorbidity burden, and health care utilization rather than intrinsic susceptibility [[17], [18], [19], [20]]. The comparatively lower prevalence of chronic comorbidities in our population, relative to some cohorts in the literature, may reflect differences in case-mix, referral patterns, or local ICU admission criteria (Figure 1). Nevertheless, the near-universal presence of prolonged ICU stays and invasive supportive care highlights the central role of health care exposure as a primary driver of acquisition.

The exceptionally high proportion of patients with extended ICU hospitalization in our study reinforces the concept of *C. auris* as a pathogen of the hospital ecosystem. Prolonged stays increase cumulative exposure to contaminated surfaces, medical devices, and health care personnel while amplifying selective pressure through repeated and prolonged courses of antimicrobial therapy [21]. This dynamic likely contributes to colonization and subsequent progression to invasive disease. The substantial cumulative ICU days observed in our cohort further emphasize the importance of environmental decontamination and strict adherence to contact precautions as cornerstones of outbreak control (Table 1).

Surgical exposure has been variably reported as a risk factor for *C. auris* infection. The lower proportion of major surgical procedures in our cohort compared with reports from South Asia may reflect differences in patient populations and institutional case-mix [22]. Nonetheless, the diversity of surgical interventions observed suggests that the risk is not procedure-specific but rather related to the cumulative burden of invasive devices, breaches in skin and mucosal barriers, and postoperative critical care requirements.

The pattern of antecedent antimicrobial exposure observed in our study aligns with a growing body of evidence linking extensive use of broad-spectrum antibiotics to fungal overgrowth and dysbiosis, thereby facilitating opportunistic infections. The high utilization of carbapenems, glycycline-class agents, and last-line antibiotics such as polymyxins underscores the severity of illness in this population and reflects the complex interplay between bacterial resistance and secondary fungal emergence. These findings highlight the need for integrated antimicrobial and antifungal stewardship programs that address the collateral impact of antibacterial therapy on fungal epidemiology.

Antifungal resistance remains one of the defining and most concerning features of *C. auris* (Table 2). Our observation of universal amphotericin B resistance contrasts sharply with many international reports and raises important methodological and epidemiologic considerations. As noted in previous comparative studies, automated systems such as VITEK 2 may yield discordant results compared with reference broth microdilution methods, particularly, for amphotericin B. Although it is surprising that all *C. auris* strains are resistant to amphotericin B, there are publications suggesting that amphotericin B MIC values are unreliable [23]. This underscores the necessity of confirmatory testing in reference laboratories, especially when unusually high resistance rates are observed, to avoid misclassification that could influence clinical decision-making and surveillance data.

Methodologically, the reliance on an automated antifungal susceptibility testing platform represents a pragmatic strength and a potential source of systematic bias. Although automated systems facilitate rapid, routine testing in high-throughput clinical laboratories, several comparative studies have demonstrated variable concordance with reference broth microdilution, particularly, for amphotericin B and echinocandins [[23], [24], [25], [26]]. In this context, the uniformly elevated amphotericin B resistance observed in our isolates should be interpreted cautiously and may warrant confirmatory testing in reference laboratories. The absence of molecular typing further limits the ability to distinguish between clonal spread and independent acquisition events, underscoring the need for integration of genomic surveillance into future institutional and national monitoring frameworks.

At the same time, the persistence of fluconazole resistance in the majority of isolates in our cohort is consistent with clade-specific resistance mechanisms described in population-based genomic studies [23,27,28]. The evolutionary trajectory of *C. auris*, shaped by decades of azole exposure in clinical and agricultural settings, highlights the global and ecologic dimensions of antifungal resistance. The convergence of environmental, agricultural, and clinical selective pressures may partially explain the parallel emergence of resistant clades across disparate geographic regions.

Therapeutically, the favorable susceptibility profile of our isolates to echinocandins and the high rate of caspofungin use in our cohort are concordant with current international guidelines [25]. The successful outcome observed in the patient treated with voriconazole illustrates that individualized therapy based on susceptibility testing can be effective in selected cases. However, the recognition of a substantial proportion of cases only after patient death in our study underscores the critical importance of early diagnosis. This finding reinforces the clinical reality that even optimal antifungal therapy cannot mitigate mortality if recognition of infection is delayed.

From a clinical perspective, the substantial proportion of cases in which *C. auris* was identified only after patient death reflects a critical problem in early diagnostic recognition. This finding underscores the limitations of conventional culture-based workflows in critically ill populations, where delays in species-level identification may preclude timely initiation of targeted antifungal therapy. The implementation of rapid diagnostic tools, including MALDI-TOF MS–based screening algorithms and molecular assays for high-risk patients, could shorten time to diagnosis and facilitate earlier therapeutic intervention, particularly, in settings with a rising institutional burden of *C. auris* (Table 1).

Mortality rates in our cohort were comparable to those reported in long-term care facilities and regional studies from the Middle East and Central Asia [11,26]. Although this consistency suggests that our outcomes are reflective of the broader epidemiologic context, it also highlights the persistent lethality of *C. auris* candidemia despite advances in critical care and antifungal therapy. The multifactorial nature of mortality—encompassing host vulnerability, severity of underlying illness, delays in diagnosis, and limitations of available antifungal agents—complicates efforts to attribute outcomes to any single determinant.

The increasing annual incidence observed in this institution also has direct implications for infection prevention strategies (Figure 2). Evidence from outbreak investigations suggests that routine environmental cleaning protocols may be insufficient for the eradication of *C. auris*, given its ability to persist on dry surfaces and withstand commonly used disinfectants. In response to the rising case burden, targeted interventions such as enhanced contact precautions, cohorting of colonized or infected patients, and the use of disinfectants with documented fungicidal activity against *C. auris* should be prioritized. Regular auditing of adherence to hand hygiene and environmental decontamination practices, coupled with staff education programs, may further mitigate institutional transmission.

From a public health perspective, the limited number of studies conducted in Türkiye underscores a critical knowledge gap. The work by Erganis et al. [29] on disinfectant susceptibility represents an important step toward understanding environmental persistence and control measures. Building on this foundation, future national surveillance initiatives integrating clinical, microbiological, and environmental data are essential to map transmission pathways and inform evidence-based infection prevention strategies.

At the national level, the limited availability of standardized reporting systems for *C. auris* hampers the ability to accurately estimate disease burden and identify inter-facility transmission networks. The integration of hospital-based surveillance data into centralized public health registries could facilitate early detection of regional clusters and support coordinated outbreak responses. Furthermore, harmonization of susceptibility testing methodologies and reporting standards across institutions would improve the comparability of resistance data and inform evidence-based updates to national antifungal stewardship policies.

### Limitations

This study has several limitations that should be considered when interpreting the findings. First, its single-center and retrospective design limits the generalizability of the results to other institutions and health care systems with different infection control practices and antifungal stewardship policies. Second, the relatively small sample size (n = 31) reduces the statistical power to identify independent predictors of mortality, resistance patterns, or treatment outcomes.

In studies comparing the reference broth microdilution method with the VITEK 2 system for antifungal susceptibility testing of *C. auris*, poor categorical agreement—particularly, for amphotericin B—has been reported, and the importance of confirming automated system results with reference methods has been emphasized [23,30]. This may have contributed to potential overestimation of amphotericin B resistance and underestimation of fluconazole resistance, thereby affecting the interpretation of local resistance epidemiology. In addition, molecular typing and clade analysis were not performed, precluding assessment of transmission dynamics, outbreak-relatedness, and the possible presence of specific resistance-associated mutations.

The absence of routine screening cultures for colonization represents a significant challenge to early detection and outbreak prevention. As a result, colonized but asymptomatic patients and environmental reservoirs may have gone unrecognized, limiting the ability to implement timely infection control interventions. Furthermore, the high proportion of patients in whom *C. auris* was detected postmortem constrained the evaluation of treatment effectiveness and time to therapy as determinants of outcome.

Finally, detailed data on environmental contamination, health care worker carriage, antifungal drug exposure prior to ICU admission, and antifungal serum levels were not available, which may have limited a comprehensive assessment of risk factors for acquisition, persistence, and therapeutic failure.

## Conclusion

In conclusion, this single-center experience identifies *C. auris* as a persistent and clinically significant cause of bloodstream infections among critically ill patients, associated with substantial antifungal resistance and high in-hospital mortality. The predominance of fluconazole resistance and the elevated amphotericin B MIC values observed further reinforce the central role of echinocandins as first-line therapy in alignment with international guidelines.

Beyond therapeutic challenges, our findings underscore that delayed recognition of colonization and infection remains the principal obstacle to effective control. Limited routine screening, diagnostic delays, and restricted access to reference susceptibility testing and molecular epidemiology substantially hinder timely intervention. In high-risk settings, such as intensive care units, passive detection is insufficient.

Effective containment requires a proactive and integrated strategy: implementation of routine admission screening in high-risk units, strict contact precautions, enhanced environmental decontamination protocols targeting persistent reservoirs, standardized antifungal susceptibility testing using reference broth microdilution methods, and rapid diagnostic workflows. These measures must be supported by robust antifungal stewardship programs and continuous staff training to ensure sustained adherence.

Future multicenter, prospective investigations incorporating molecular typing, environmental surveillance, and standardized reference susceptibility testing are essential to clarify transmission dynamics and resistance mechanisms. Establishing national reference laboratories capable of performing broth microdilution and molecular typing is urgently needed to strengthen surveillance capacity and guide evidence-based therapeutic and infection control strategies against this emerging global health care threat.

## Declaration of competing interest

The authors have no competing interests to declare.
